# Good documentation practice in clinical research

**DOI:** 10.4103/2229-3485.80368

**Published:** 2011

**Authors:** Chitra Bargaje

**Affiliations:** *Department of Clinical Trials and Safety, Global Quality and Regulatory Compliance, Bristol Myers Squibb, Mumbai, India*

**Keywords:** ALCOA, documentation, source, training

## Abstract

One of the most common inspection findings in investigator site inspections is lack of reliable, accurate and adequate source documentation. This also happens to be the most common pitfall identified during sponsor audits. The importance of good documentation practice needs to be emphasized to investigator sites to ensure that the study results are built on the foundation of credible and valid data. This article focuses on the key principles of good documentation practice and offers suggestions for improvement.

## INTRODUCTION

Inadequate/inaccurate case histories form the second most commonly cited deficiency in US-FDA inspections of clinical investigator sites.

Similarly, source documentation issues ranked 5th among the top 10 findings from European Medicines Agency (EMA) inspections of investigator sites in 2009[1] and in some instances the findings were classified ‘critical’. Not surprisingly, clinical trial monitors and auditors also report documentation issues as a frequent area of GCP concern.

I would like to share an experience at a recent investigator site audit.

During the audit opening meeting we were informed that all the source data is on paper and no electronic documentation is used. The site was actually using MS word to document the data collected during the study. In normal practice the site did not use MS word to generate medical records. This method was adopted *only* for clinical trial subjects. For the trial subjects there were no other hand-written progress notes which the site would normally use for routine patients.

There were two underlying potential issues here:

- First, the site was following a different practice for documenting progress for clinical research subjects. Were the subjects’ records missing any elements of standard care because of the deviation from routine practice?
- Second, the site thought they had no electronic documentation, although MS word was used to record all subject data.

This example, illustrates a common occurrence in clinical trial research where a lack of understanding of basic GCP principles may have a negative impact on the quality of the study.

## WHAT IS THE PURPOSE OF SOURCE DOCUMENTATION?

To understand the importance of good source documentation we should first review the purpose of source documentation. The most important purpose of source documentation in a clinical trial is to reconstruct the trial as it happened. It should enable an independent observer to reconfirm the data. Documentation should be such that it is able to provide audit trail to permit investigation if and when required.

Source documentation is the medical record of the subject before, during and after the trial.

It is the tool which confirms the eligibility criteria of the subject in the given trial.

It documents the progress of the subject from consenting till the subject completes the study. It records the accountability of the investigational product dispensed, consumed and returned by the subject. It serves as the complete medical record of the subject as the reference to the treating physician at any point of time.

Finally it forms a strong foundation for the data that gets transcribed into a CRF which ultimately gets translated into a clinical study report.

Irrespective of clinical trial, accurate documentation supports the fundamental principle of protecting subject’s rights, safety and well-being.

There can not be two thoughts to emphasize the need for reliable and quality documentation.

## PRINCIPLES OF GOOD DOCUMENTATION PRACTICE

So, what does it mean when we say ‘Good Documentation’ and how do we practice it?

Any basic training in clinical research will definitely include these phrases:

‘What is not documented is not done!’

‘Document what is done as well as what is not done!’

Roots of good documentation principles are in the ICH-GCP where source data and source document is first defined.

### ICH E6 1.51 source data

All information in *original* records and *certified copies* of original records of clinical findings, observations, or other activities in a clinical trial necessary for the reconstruction and evaluation of the trial. Source data are contained in source documents (original records or certified copies).

The words in *italics* describe some inherent qualities of source data.

### ICH E6 1.52 source documents

Original documents, data and records (e.g., hospital records, clinical and office charts, laboratory notes, memoranda, subjects’ diaries or evaluation checklists, pharmacy dispensing records, recorded data from automated instruments, copies or transcriptions certified after verification as being accurate copies, microfiches, photographic negatives, microfilm or magnetic media, X-rays, subject files, and records kept at the pharmacy, at the laboratories and at medico-technical departments involved in the clinical trial).

This definition describes the various types of documents which collectively form the source document.

Key attributes for good documentation were first described by US-FDA in the form of ALCOA -attributable, legible, contemporaneous, original and accurate. These are also adapted by World Health Organization (WHO). These criteria evolved with time. EMA has added some more ‘letters’ to describe qualities of good source documentation particularly for electronic documentation.[2–4]

Let‘s look at these attributes described by different authorities collectively.

### Attributable

It should be clear who has documented the data.

### Legible

Readable and signatures identifiable.

### Contemporaneous

The information should be documented in the correct time frame along with the flow of events. If a clinical observation cannot be entered when made, chronology should be recorded. Acceptable amount of delay should be defined and justified.[4]

### Original

Original, if not original should be exact copy; the first record made by the appropriate person. The investigator should have the original source document.

### Accurate

Accurate, consistent and real representation of facts.

### Enduring

Long-lasting and durable.

### Available and accessible

Easily available for review of treating physicians and during audits/inspections. The documents should be retrievable in reasonable time.

### Complete

Complete till that point in time.

### Consistent

Demonstrate the required attributes consistently.

### Credible

Based on real and reliable facts.

### Corroborated

The data should be backed up by evidence.

Interestingly, it should be noted that the Drug Controller General India (DCGI) would emphasize on the *condition* in addition to the completeness, legibility and accessibility of investigator source data file as noted in DCGI’s guidance document for inspections.[5] My understanding of ‘condition’ is the state of the source documents, in terms of filing, storing and readability.

The degree to which the data fulfills the data quality criteria establishes acceptability of the data. It also determines the degree of excellence of the data quality. Qualities like consistency, credibility and corroboration help establish data integrity along with the data quality.

These are the expectations from clinical trial documentation however in reality many issues are observed in terms of quality of source documentation.

## COMMON FINDINGS WITH RESPECT TO SOURCE DOCUMENTATION

*‘Failure to maintain adequate and accurate case histories that record all observations and other data pertinent to the investigation on each individual administered the investigational drug or employed as a control in the investigation’* is cited in 6 out of the 10 warning letters issued by US-FDA to clinical investigators in 2010.[6]

At one investigator site source documents were not available because the computer *‘crashed’*. So in the absence of availability, adequacy of the records could not be evaluated. The investigator was warned for *‘failure to retain records required to be maintained for the required timeframe per regulations’*.

I would like to highlight some of the findings from the warning letters in detail here. These findings give an idea of regulatory expectations and lacunae in documentation noted during inspections. I am sure readers would be able to relate to some of these findings with their personal experience.

- Eligibility criteria could not be confirmed. For e.g., (a)IVRS user manual states “Complete call worksheets prior to contacting the IVRS; then file completed worksheets with each subject’s source documentation.” The IVRS worksheets were not kept in the subjects’ files or maintained at the site and as such it could not be confirmed that patients were stratified in the right arm and received the medication they were assigned to. (b) All the items in the exclusion criteria checklist are checked except for the exclusion criterion related to the history of thrombocytopenia, including heparin-induced thrombocytopenia, or a platelet count <100,000 cells/microliter. In the absence of lab report this exclusion criteria could not be confirmed on the basis of the incomplete checklists.
- Multiple records for *same data* points making it unable to determine which served as the accurate source record, for e.g., multiple versions of visual analog scales completed for same visit with different values.
- Discrepancies in records to confirm primary efficacy endpoint of the study, for e.g., the total administered dose of morphine, as reflected in hospital records was different from the Case Report Form. The primary efficacy endpoint of the protocol was to measure the reduction in the requirement for morphine use in the 24 hours following surgery measured by total morphine usage compared to placebo.
- Clinical significance for out of range lab values not documented on the lab reports or conflicting information found in the source documentation-e.g., significant high glucose value marked as clinically nonsignificant on the lab report although the subject was referred to for primary physician for further follow-up.
- Missing pages from subject interview scales, numerous unexplained corrections months after the initial entries and conflicting information; incorrect subject identifiers, incorrect date e.g., same date on screening visit, visit week 1 and week 4.
- Numerous AEs not reported in CRFs, delays in transcribing data in CRFs, discrepancies between source and the CRF. Lack of timely reporting of AEs in eCRFs jeopardizes subject safety and reliability and integrity of data captured at the site.
- Incorrect/incomplete documentation regarding the disposition of drugs-dates, quantity and use by subjects.

Although some of these issues may appear minor prima facie such as some checkboxes not checked, a lab report not marked for significance for out of range value, some discrepancies in source and CRF, unexplained corrections, these issues point toward lack of understanding of good documentation requirements. For an independent observer such data would fail to provide confidence and assurance of data quality and safety of the subjects enrolled. The data may be deemed unfit for use. All exposure of patients to new drugs and the efforts and time spent by the investigator team would be wasted.

Systematic deficiencies in documentation can lead to questions about the integrity of the data, potentially resulting in health authority decisions to exclude the data from analysis.

In essence, we can definitely say that the quality of documentation can make or break the study at a given site.

## WHAT ARE THE POSSIBLE ROOT CAUSES FOR REPEATED DEFICIENCIES IN SOURCE DOCUMENTATION?

Clinical research documentation involves a variety of documents from various sources and is often completed by several people. Thus rendering this process to be complicated and posing challenges to meet requirements. Moreover clinical research happens over a long period of time which adds to the challenge of maintaining continuity in the documentation practice.

Inadequacies in documentation could be the result of lack of training and experience in good understanding of clinical research and documentation requirements. As a result the principal investigator (PI) and staff may continue documentation per the routine medical practice. In India, the documentation in routine medical practice may not be as extensive as what would be expected for clinical research.

Additional unmonitored medical records are discovered at the time of audits/inspections. Such as: Diaries of coordinator, inpatient records of the hospital, electronic records, etc., for the simple reason that the staff does not realize that these form a part of source record. These unmonitored records may have important data which do not find its way to the CRF. This would have an impact on the availability of important information in CRFs. Reliability and integrity of data might me affected as a result.

In many FDA warning letters one can observe that inadequate case histories, consenting or drug disposal records are often attributed to the lack of investigator’s supervision in ensuring compliance. The PI delegates responsibilities to the study team and may not provide adequate time to review the source data due to lack of time or commitment. The study documentation is completely left on the shoulders of study coordinator’s.

Various tools are used for data collection. At times sponsor provides source document worksheets to ensure complete documentation. If these worksheets are not designed accurately to align with the protocol and CRF source data quality is directly impacted. These worksheets are often completed as checkboxes without any additional notes, comments or supporting documents. Source document worksheets sometimes also result in multiple records. The sites continue to maintain the clinical practice routine documentation and worksheets are completed in addition for the study. As such, these worksheets are no longer a primary source and thus no source document.

Workload of the existing staff can be another important reason leading to poor documentation. This may cause errors like source data for one subject entered in another subject record, pages misfiled, use of incorrect consent forms and similar issues.

Study coordinator/PI work with various sponsors/CROs at a time. These different sponsors/CROs communicate different level of expectations regarding source documentation. If the site is not experienced enough and they do not have a standard procedure to follow they may get confused with variations in guidance they receive. This may negatively impact the quality of data.

Certain technical inadequacies may also lead to poor source documentation. For e.g., the ECG machine is old and does not print the date, time and subject identifiers, printer or fax machine does not work. If the fax is not working it may result in not receiving important data i.e., lab reports, data queries, investigational product allocation confirmations, SAE transmission confirmations, etc. Important email correspondence with sponsor/CROs if not printed and archived may get lost.

## HOW CAN THE DOCUMENTATION BE IMPROVED?

Based on the various causes noted above, I would like to offer some suggestions to improve the quality of source documentation at sites.

- PI should delegate responsibilities to staff adequately trained in protocol and GCP. Particular training should be provided on ALCOA and other good documentation practice requirements. Medical decisions should be delegated to medically qualified staff. Training of site staff should be repeated at defined frequency. New hires should be adequately trained before trial participation.
- PI should commit for involvement, and supervision throughout the entire duration of the study. There should be an agreed and documented procedure for PI to ensure supervision of the study by meetings with site staff, monitors; review of documentation, timely resolution of medical, ethical or GCP issues. The PI or designated subinvestigators should validate the medical data. The PI should also supervise the work of SMO staff and external facilities if used. In case there are performance issues with SMO staff or external facility PI should immediately inform the supervisor as well as sponsor.
- Site should develop a SOP for good documentation. This SOP should be shared with the sponsor/CRO and agreed upon before the start of the trial. This SOP should address aspects including but not limited to consenting process, verifying eligibility, use of right tools such as diaries, source document worksheets, OPD papers, copies of prescriptions, etc; ways to avoid multiple records and in case of multiple records should define the source for the study, method of corrections, review of safety labs and other reports. Documented procedure at site level should encompass management, maintenance, archival and retrieval of source documentation. Sites should have measures for continuous improvement and maintaining high-quality data. Sites should develop process for quality control.
- Before the trial commences all technical aspects such as for e-CRFs, fax, printers, etc. should be clarified and issues resolved. In case of any difficulties during the trial, sponsor should be informed and back-up plans agreed upon till the issue is resolved. In case when original lab records or investigational records are sent to central location for assessment, process should be in place to ensure a duplicate copy or certified copy is available in the site source records.
- Sponsor/CRO also plays an important role in ensuring quality of source documentation. Sponsor/CRO should ensure PI’s commitment and involvement throughout the study. Sponsor/CRO should assess the site’s documentation practice during pre-study visit and during the study; provide training to the site staff to reinforce expectations. Time spent effectively during pre-study evaluation on source documentation would help a great deal to minimize documentation issues later. The source data and their respective capture methods should be clearly defined prior to trial recruitment i.e. in the protocol or study specific source data agreement.

## CONCLUSIONS

Source documentation should demonstrate the ALCOA and other attributes as described by regulatory authorities and GCP. Source documentation related findings are the most commonly cited during inspections and audits. PI’s commitment and involvement in the trial makes a huge difference. Efforts to train the sites, understand the sites practices right from the pre-study visit and continuous monitoring and training would definitely help in improving and maintaining the quality of site source documentation practices.

Ultimately the source document should speak for itself. It should narrate the medical journey of the patient as it happened to an independent observer-an auditor or inspector and thus form a strong foundation for a good clinical research.
